# Audiotactile interactions in the mouse cochlear nucleus

**DOI:** 10.1038/s41598-021-86236-9

**Published:** 2021-03-25

**Authors:** Josephine Ansorge, Calvin Wu, Susan E. Shore, Patrik Krieger

**Affiliations:** 1 Department of Systems Neuroscience, Faculty of Medicine, Ruhr University Bochum, Universitätsstraße 150, 44780 Bochum, Germany; 2 Department of Otolaryngology, Kresge Hearing Research Institute, University of Michigan, Ann Arbor, MI USA; 3 Biomedical Engineering, University of Michigan, Ann Arbor, MI USA; 4 Molecular and Integrative Physiology, University of Michigan, Ann Arbor, MI USA

**Keywords:** Auditory system, Sensory processing, Neuroscience, Whisker system

## Abstract

Multisensory integration of auditory and tactile information occurs already at the level of the cochlear nucleus. Rodents use their whiskers for tactile perception to guide them in their exploration of the world. As nocturnal animals with relatively poor vision, audiotactile interactions are of great importance for this species. Here, the influence of whisker deflections on sound-evoked spiking in the cochlear nucleus was investigated in vivo in anesthetized mice. Multichannel, silicon-probe electrophysiological recordings were obtained from both the dorsal and ventral cochlear nucleus. Whisker deflections evoked an increased spiking activity in fusiform cells of the dorsal cochlear nucleus and t-stellate cells in ventral cochlear nucleus, whereas bushy cells in the ventral cochlear nucleus showed a more variable response. The response to broadband noise stimulation increased in fusiform cells and primary-like bushy cells when the sound stimulation was preceded (~ 20 ms) by whisker stimulation. Multi-sensory integration of auditory and whisker input can thus occur already in this early brainstem nucleus, emphasizing the importance of early integration of auditory and somatosensory information.

## Introduction

In the auditory system, integration of information from the cochlea with information from other sensory modalities begins at the earliest processing stages in the cochlear nucleus^1–6^. Anatomical studies demonstrate that several somatosensory structures in the brainstem provide inputs to the cochlear nucleus (CN). These regions include the dorsal column nuclei, consisting of the gracile^7^ and cuneate^8,9^ nuclei that receive proprioceptive and somatosensory inputs from the lower and upper body, respectively. Projections to the cochlear nucleus also arise from the spinal trigeminal nucleus^10–14^ that conveys touch sensation from the face. Projections from these regions form mossy fibre inputs that synapse onto granule cells in the cochlear nucleus granule-cell domain, as well as onto dendrites of ventral cochlear nucleus (VCN) bushy cells^15^ and D-stellate cells^16^. The D-stellate cells inhibit fusiform cells in dorsal cochlear nucleus (DCN)^17–19^. The parallel fibre axons of granule cells in turn provide excitatory synaptic input to DCN cartwheel and fusiform cells. The presence of somatosensory inputs to a primary auditory nucleus such as the cochlear nucleus is intriguing in the context of the role of the pinnae and neck in generating sound localization cues^16^, as well as the suppression of self-generated signals^20^. Correct interpretation of this information requires integration of auditory signals with somatosensory and proprioceptive signals conveying information about head and pinna position. Studies in cats and guinea pigs^6,21–23^ have demonstrated that direct electrical stimulation of brainstem somatosensory nuclei evokes neuronal responses in the DCN and VCN^15,21,24^. These data suggest that activation of CN granule cells by these somatosensory inputs excites fusiform cells and provides feedforward inhibition to fusiform cells through the inhibitory interneurons, the cartwheel cells. The association between sound and whisker stimulation may be a consequence of similar encoding mechanisms: both senses process information that produces mechanical displacements of tissue (i.e., the basilar membrane for auditory and the skin for somatosensory) and are processed in frequency based codes in the cerebral cortex^25^. Rodents use a set of roughly 30 whiskers on each side of the snout, palpating surfaces through a 5–20 Hz forward‐backward motion known as “whisking”^26,27^. Whisker‐mediated object identification can thus be used as a model to learn more about the mechanisms of multisensory processing and the transformation of this processing to a behavioural output. In the present in vivo electrophysiology study, spiking activity was measured in spike-sorted single units, and audiotactile interaction in the mouse cochlear nucleus was investigated using whisker stimulation in combination with sound. The results suggest that whisker stimulation can modify the sound-evoked spiking activity in the cochlear nucleus.

## Results

### Cell type classification

**Figure 1:**
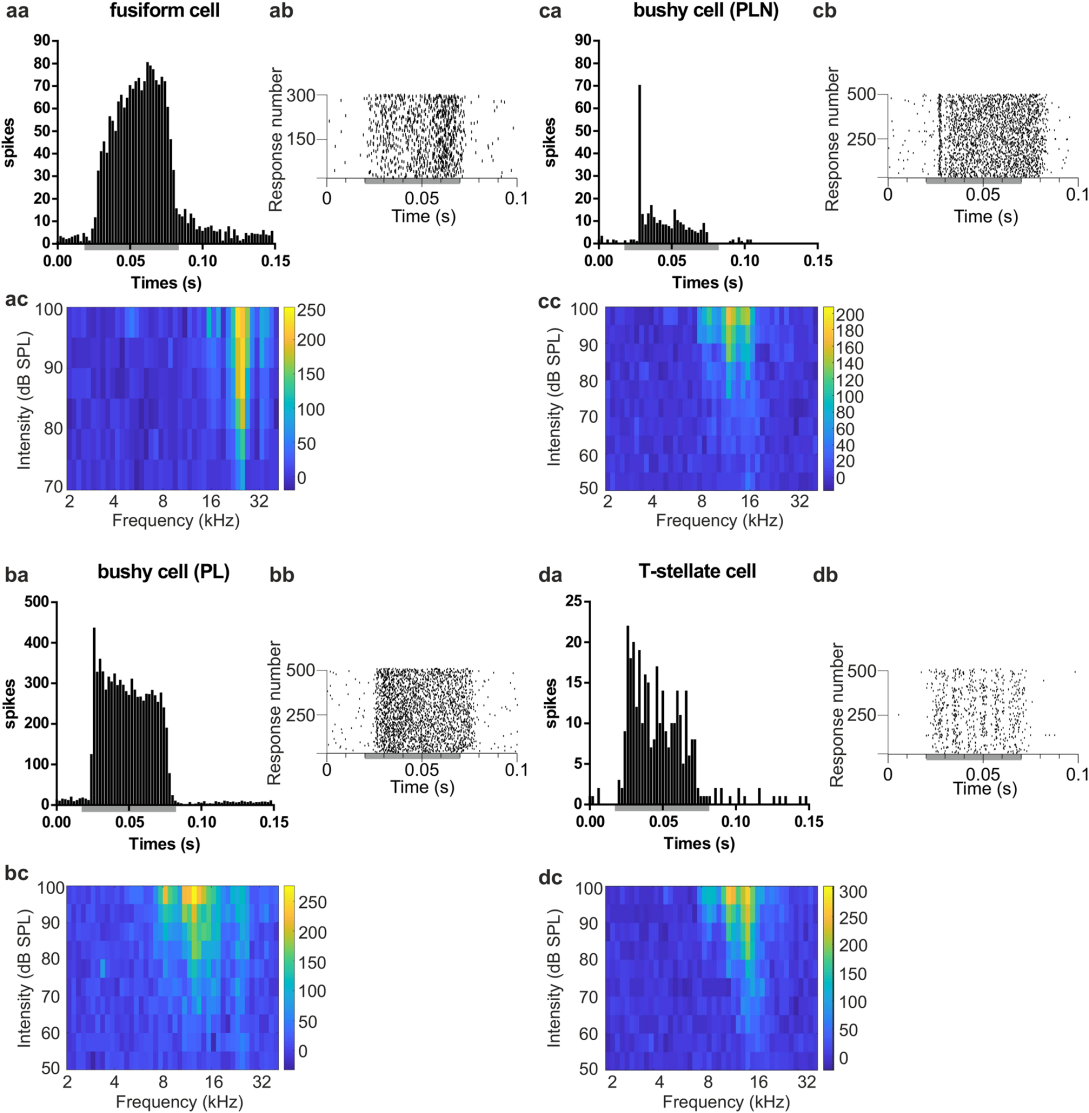
Cell classification. The neurons recorded in the cochlear nucleus were classified based on their characteristic response profile. (**aa**) Post-stimulus time histogram (PSTH, 2 ms bin, 300 sweeps) of a spike-sorted unit from a fusiform cell in DCN recorded during best-frequency tone stimulation (24,261 Hz) at 20 dB above threshold. The PSTH shows the characteristic build-up response. (**ab**) Raster plot of the fusiform cell from *aa*. (**ac**) Receptive field (frequency intensity response pattern; colour code is spike rate in 50 ms from 10 sweeps) for the same fusiform cell. (**ba**) PSTH (2 ms bin, 500 sweeps) of a spike-sorted unit from a primary-like bushy cell (PL) in VCN recorded during best-frequency tone stimulation (12,130 Hz). (**bb**) Raster plot of the primary-like bushy cell from *ba*. (**bc**) Receptive field (colour code is spike rate in 50 ms from 10 sweeps) for the primary-like bushy cell in *ba*. (**ca**) PSTH (2 ms bin, 500 sweeps) of a spike-sorted unit from a primary-like-with-notch bushy cell (PLN) in VCN, recorded during best-frequency tone stimulation (10,560 Hz). (**cb**) Raster plot of the PLN bushy cell from *ca*. (**cc**) Receptive field of the primary-like bushy cell with notch. (**da**) PSTH (2 ms bin, 500 sweeps) of a spike-sorted unit from a t-stellate cell in VCN, recorded during best-frequency tone stimulation (14,934 Hz). (**db**) Raster plot of the spike-sorted t-stellate cell from *da*. (**dc**) Receptive field (colour code is spike rate in 50 ms from 10 sweeps) for the t-stellate cell. In the panels showing PSTH and raster plots, the grey bar is the 50 ms tone stimulation.

Neurons in the mouse CN were classified based on their characteristic post-stimulus time histogram (PSTH) at best frequency (BF; as determined from a receptive-field analysis) 20 dB above threshold, as well as receptive-field types (Fig. 1) and the electrode location in DCN or VCN, verified by the electrode tract. Forty-five cells were classified as putative fusiform cells (pause/build-up temporal pattern) with type III and type I-III receptive fields in DCN (Fig. 1aa-ac) and 49 cells as putative VCN bushy cells (primary-like (n = 42; Fig. 1ba-bc) and primary-like with notch (n = 7; Fig. 1ca-cc)^28^). Furthermore, 10 cells in VCN were classified as putative t-stellate cells (5 transient choppers and 3 sustained choppers, 2 undefined, Fig. 1da-dc) based on their coefficients of variation^29^. Tone stimulation increased spiking in fusiform cells 4.1 ± 2.2 ms after stimulus onset, in bushy cells after 4.3 ± 1.6 ms and in t-stellate cells after 4.9 ± 1.8 ms.

### Whisker-evoked responses in fusiform cells in DCN and bushy cells in VCN

**Figure 2:**
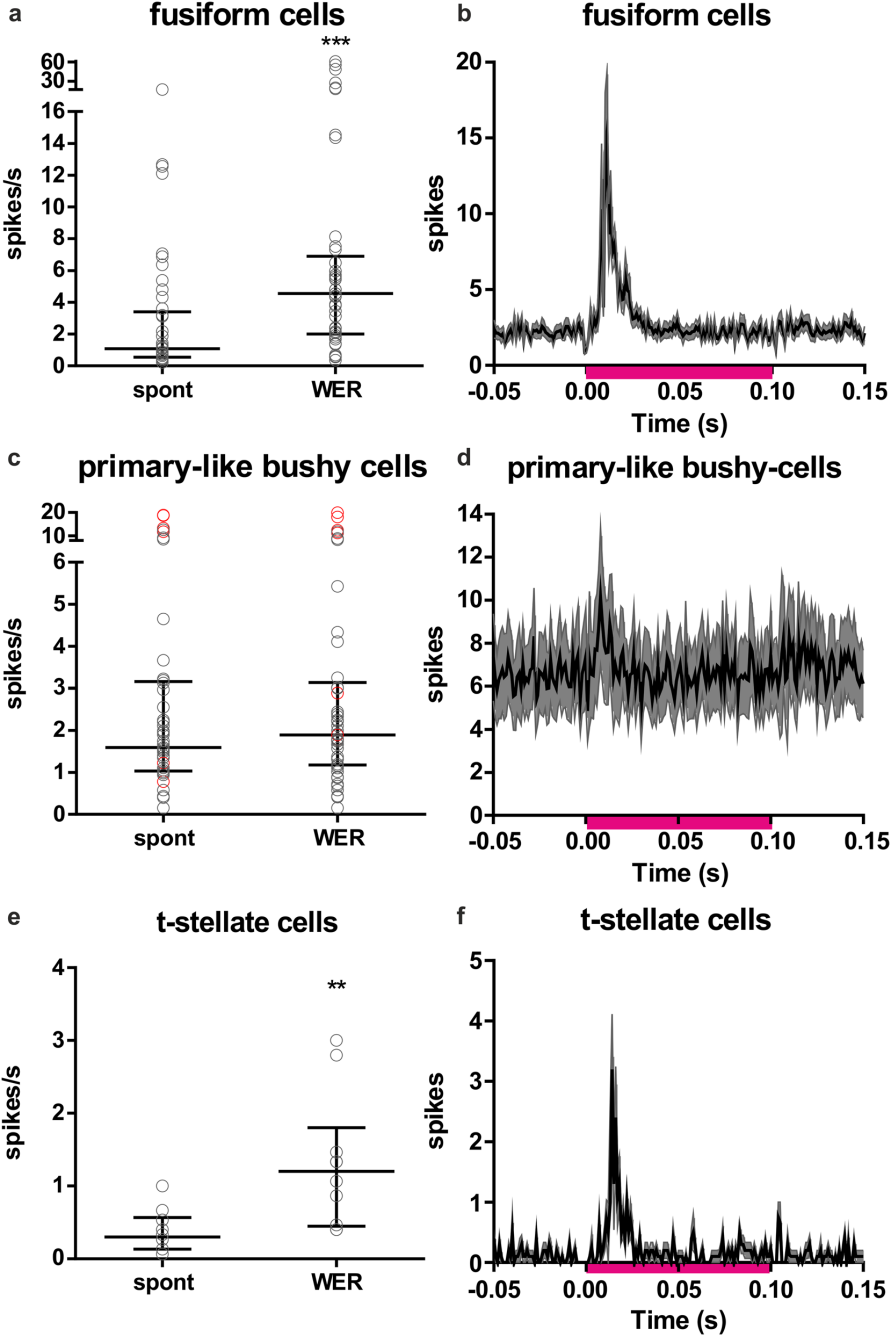
Whisker-evoked responses in fusiform cells, primary-like bushy cells and t-stellate cells. (**a**) Spontaneous spiking (spont) and the whisker-evoked response (WER) in fusiform cells. Whisker deflections increased median spiking in fusiform cells from 1.09 spikes/s to 4.56 spikes/s with whisker stimulation (n = 45; p < 0.0001, Wilcoxon matched pairs signed rank test). (**b**) The averaged PSTHs (1 ms bins) of all recorded fusiform cells. The sum of 1000 whisker-evoked stimulations were calculated for each cell, and then the average from these sums was calculated and plotted with a SEM confidence band. (**c**) Spontaneous spiking (spont) and the whisker-evoked response (WER) in bushy cells. Black circles are primary-like bushy cells (PL) and red circles are primary-like-with-notch bushy cells (PLN). Whisker deflections did not significantly change the median spiking activity (spont. 1.59 spikes/s; whisker stimulation 1.89 spikes/s; p = 0.1005, Wilcoxon matched-pairs signed rank). (**d**) The averaged PSTHs (1 ms bins) of all recorded primary-like bushy cells (PL and PLN). The sum of 1000 stimulations were calculated for each cell, and then the average from these sums was calculated and plotted with SEM confidence band. (**e**) Spontaneous spiking (spont) and the whisker-evoked response (WER) of t-stellate cells (n = 10). Whisker deflections increased spiking from 0.30 spikes/s to 1.20 spikes/s (n = 10; p = 0.0020, Wilcoxon matched-pairs signed rank test). (**f**) The averaged PSTHs (1 ms bins) of all recorded t-stellate cells. The sum of 1000 stimulations were calculated for each cell, and then the average from these sums was calculated and plotted with a SEM confidence band. In panels b, d & f the pink bar shows the time of whisker stimulation (0 to 0.10 s). In panels a, c & e the error bar indicates the median and the inter-quartile range (IQR).

The whiskers on the mouse whisker pad were deflected (1000 deflections at 5 Hz) using a magnetic stimulation system, and spiking activity was measured in the CN using multi-channel silicon probes. In DCN fusiform cells whisker stimulation evoked increased spiking activity above the spontaneous rate. The increase in spiking started 6.0 ± 1.8 ms after whisker movement. Calculated over the full spike-sorted cell population (n = 45 cells; 8 animals) whisker stimulation increased median spiking from 1.09 spikes/s [IQR: 0.55–3.40] to 4.56 spikes/s with whisker stimulation [IQR: 2.00–6.90] (p < 0.0001, Wilcoxon matched-pairs signed rank test; time window for calculating spikes was 50 ms/sweep; 1000 sweeps were recorded; Fig. 2a, b).

In primary-like bushy cells in VCN the spontaneous activity (n = 49 cells; 5 animals) did not change with whisker stimulation (Fig. 2c, d). The median spontaneous activity was 1.59 spikes/s [IQR: 1.03–3.16], and with whisker stimulation 1.89 [IQR: 1.18–3.14] (p = 0.1005, Wilcoxon matched-pairs signed rank). Splitting the cell population into primary-like (PL) and primary-like-with-notch (PLN) did not change the result. (PL (n = 42) spontaneous activity: 1.54 spikes/s [IQR: 0.99–2.66], whisker-evoked response (WER): 1.76 spikes/s [IQR: 1.34–2.58] p = 0.1181; PLN (n = 7) spontaneous activity: 11.75 spikes/s [IQR: 1.22–18.69], WER: 11.27 spikes/s [IQR: 1.89–18.04], p = 0.4688). It should be noted, however, that although when averaging over the entire sample there was no effect of whisker stimulation, there were individual PL cells where small effects were observed (Fig. 2d). The number of recorded t-stellate cells in VCN was comparatively low (n = 10 cells, from one animal, two different recording sites, 2 shank electrode), but there was an increase in spiking from 0.30 spikes/s [IQR: 0.13–0.57] to 1.20 spikes/s [IQR: 0.45–1.8] when whiskers were deflected (Wilcoxon matched-pairs signed rank, p = 0.0020; IQR: 0.32–1.35) effect size: 0.63 (Fig. 2e, f). This increase could be detected 7.2 ± 2.3 ms after whisker movement.

### Whisker stimulation increased the sound-evoked response in DCN fusiform cells and VCN bushy cells

Stimulation of the ipsilateral whiskers (ipsilateral to recording site in the CN) during acoustic stimulation increased the sound-driven responses of fusiform cells and bushy cells. The magnitude of the effect was dependent on the delay between whisker stimulation and sound stimulation onset. The whisker stimulation onset shifted from 50 ms before sound (+ 50), to 20 ms after onset of sound (−20); the protocols were performed in a random order (Supplementary Table S2).

**Figure 3:**
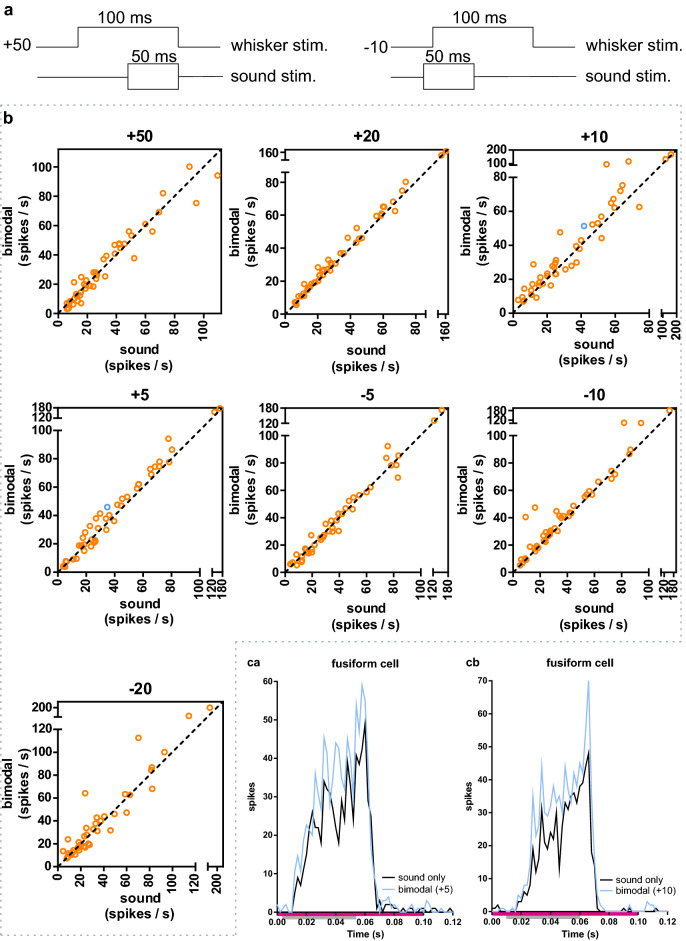
Bimodal responses in fusiform cells. (**a**) Two schematic examples of how the different stimulation protocols were performed. *Left*: A 100 ms whisker stimulation starting 50 ms before (“ + 50”) the onset of a 50 ms sound (broadband noise) stimulation. *Right*: A 100 ms whisker stimulation starting 10 ms after (“−10”) the onset of a 50 ms sound stimulation. (**b**) Bimodal (whisker and sound) response compared to only sound stimulation. Each protocol (+ 50, + 20 + etc.; see Methods) is plotted separately and each spike-sorted unit is represented with a circle. The dotted line represents a linear regression line with slope 1 (y = x). The bimodal + 20, + 10, + 5 and − 5 protocols evoked a larger response compared to only sound stimulation. See Table 1 for numbers and statistics. Blue circles (one in the + 5 and one in the +10  plot) are for the example cells in *ca-cb*. (**ca**) Example PSTH for a fusiform cell showing the response to only sound compared to the + 5 bimodal protocol. The bimodal response is increased compared to the only sound response. Pink bar shows the time of whisker stimulation (from 0 to 0.1 s); the grey bar marks the duration of the sound stimulation (from 0.005 to 0.055 s; sum of 500 stimulations each for sound and bimodal). (**cb**) Example PSTH for a fusiform cell showing the response to only sound compared to the + 10 bimodal protocol. The bimodal response is increased compared to the only sound response. Pink bar shows the time of whisker stimulation (from 0 to 0.1 s); the grey bar marks the duration of the sound stimulation (from 0.01 to 0.06 s; sum of 500 stimulations each for sound and bimodal).

In fusiform cells, the number of sound-evoked spikes increased for the + 20, + 10, + 5 and − 10 protocol (Fig. 3, Table 1 and Supplementary Fig. S1). The protocols where whisker stimulation preceded sound stimulation increased spiking with approx. 4 spikes/s (27 to 31 spikes/s) (average from the + 20, + 10, + 5 protocols). In the other protocols (+ 50, − 5, − 20) the bimodal stimulation did not significantly change the absolute evoked spikes compared to sound only stimulation. It remains to be investigated if small differences in timing, e.g. between the − 5 and − 10 protocol, relate to non-linear effects on encoding sensory information in the DCN. The bimodal response (calculated as *BI*; Fig. 6) was on average 9% smaller than the linear summation of the sound and whisker-evoked response (averaged over the + 20 and + 10, protocols; p < 0.001, Wilcoxon signed rank test, theoretical median zero).

**Table 1:** Fusiform cells in DCN. * Statistically significant changes (p < 0.01; Wilcoxon matched-pairs signed rank test, with Bonferroni correction). IQR = interquartile range. BI = bimodal integration index (mean). BI values are in bold when there was a significant change in median spiking between “sound” and “whisker + sound” stimulation. Positive BI values would show a bimodal enhancement. Bold values close to zero would show that there is no bimodal integration (just an additive effect of the whisker response). Bold negative BI values mean, that the bimodal response was larger than only sound, but not larger than the sum of sound and whisker stimulation alone. Negative BI values, without a significant difference between sound and whisker + sound, indicates that whisker stimulation alone evoked a response.

Protocol	Median (spikes/s)		BI	IQR		p-value (Bonferroni corrected)	Effect size *r*
	Sound	Whisker + sound		Sound	Whisker + sound		
+ 50	25.82	25.31	−19.81	14.16–44.13	12.84–47.30	> 0.9999	0.04
+ 20	26.47	28.36	−**10.99**	15.72–51.44	18.29–55.88	0.0007*	0.50
+ 10	25.18	28.80	−**7.14**	15.24–51.92	17.21–54.99	0.0042*	0.35
+ 5	29.77	36.11	−**12.77**	18.08–56.89	18.85–62.02	0.0021*	0.37
−5	32.30	30.19	−20.53	16.63–52.54	15.63–55.67	0.9999	0.02
−10	30.69	39.88	−**14.31**	16.86–55.27	20.85–56.88	0.0007*	0.44
−20	23.88	24.00	−16.37	14.43–47.64	14.34–46.64	0.9999	0.11

**Figure 4:**
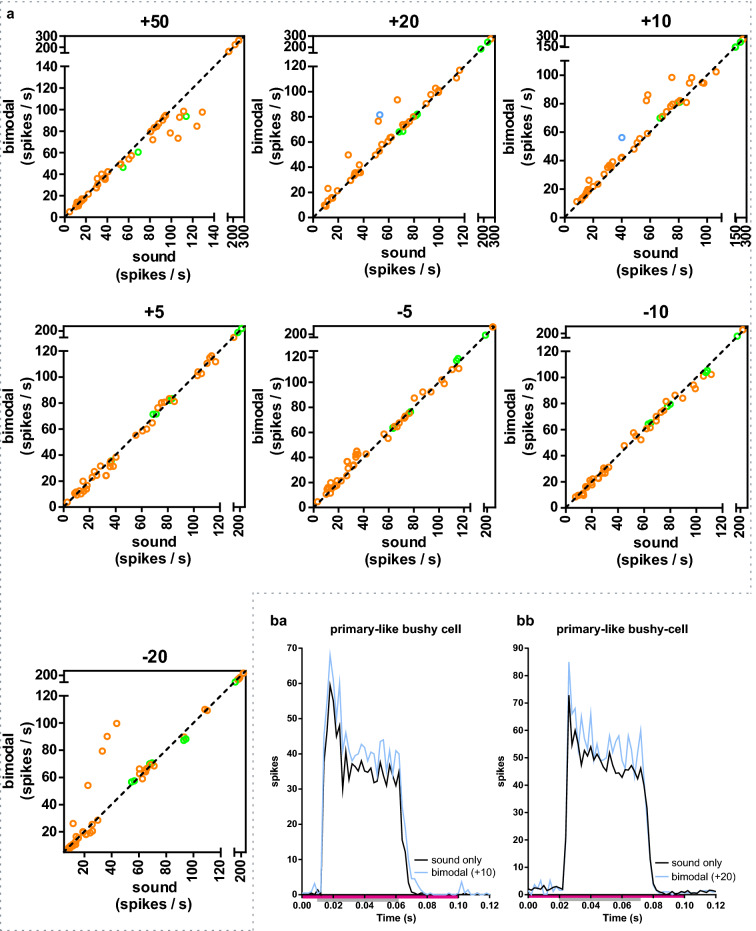
Bimodal response in bushy cells. (**a**) Bimodal stimulation compared to sound stimulation. Each protocol (+ 50, + 2 + etc.) is plotted separately and each spike-sorted unit is represented with a circle (PL: n = 42; PLN: n = 7). The dotted line represents a linear regression line with slope 1 (y = x). Orange circles (n = 42) are primary-like bushy cells and green circles (n = 7) are primary-like-with-notch bushy cells (PLNs). In PL cells the bimodal + 20 & + 10 stimulation protocols evoked a larger response compared to only sound (Table 2). Blue circles are the example cells from *ba-bb*. 5 PL bushy cells showed an increase of the bimodal response compared to the sound only stimulation with the − 20 protocol. (**ba**) PSTH for a primary-like bushy cell for the protocol + 10. The bimodal response is increased compared to the only sound response. Pink bar shows the time of whisker stimulation (from 0 to 0.10 s); the grey bar marks the duration of the sound stimulation (from 0.01 to 0.06 s; sum of 500 stimulations each for sound and bimodal). (**bb**) PSTH for a primary-like bushy cell for the protocol + 20. The bimodal response is increased compared to the only sound response. Pink bar shows the time of whisker stimulation (from 0 to 0.20 s); the grey bar marks the duration of the sound stimulation (from 0.02 to 0.07 s; sum of 500 stimulations each for sound and bimodal).

In VCN, cells were classified based on the PSTH pattern as either primary-like (PL) or primary-like-with-notch bushy cells (PLN). In PL bushy cells, the + 20 and + 10 bimodal protocols significantly increased the number of sound evoked responses during whisker stimulation (Fig. 4, Table 2 and Supplementary Fig. S1) with approx. 5 spikes/s (48 to 53 spikes/s; average from the + 20, + 10 protocols). The bimodal response of the PL bushy cells was ~ 11% larger than the linear summation of the sound and whisker-evoked response (averaged over the + 20, + 10 protocols; p < 0.001, Wilcoxon signed rank test, theoretical median zero; Fig. 6). The + 50 protocol induced a response that was ~ 5% smaller than the linear summation of the sound and whisker-evoked response, indicating that although the bimodal response was slightly larger than only sound, it was smaller than the unimodal sum.

**Table 2:** Primary-like bushy cells and t-stellate cells in VCN. *Statistically significant changes (p < 0.01; Wilcoxon matched-pairs signed rank test and Bonferroni correction). Primary-like bushy cells (PL, n = 42 cells), Primary-like-with-notch bushy cells (PLN, n = 7) and t-stellate cells (n = 10). IQR = interquartile range. BI = bimodal integration index (mean). BI values are in bold when there was a significant change in median spiking between “sound” and “whisker + sound” stimulation. Note: 5 PL bushy cells showed an increase if the bimodal response compared to the sound only stimulation with the − 20 protocol.

Protocol	Median (spikes/s)				BI	IQR				p-value (Bonferroni corrected)/effect size *r*	
	sound		Whisker + sound			Sound		Whisker + sound			
	PL	PLN	PL	PLN	PL	PL	PLN	PL	PLN	PL	PLN
+ 50	40.53	94.67	42.24	94.72	−**5.27**	21.24–89.08	80.86–162.5	20.38–83.45	79.77–164.0	0.0056*/r = 0.35	> 0.9999
+ 20	52.14	82.73	55.45	82.06	**10.15**	29.28–77.10	71.16–174.6	29.55–83.82	68.54–184.1	0.0007*/r = 0.48	> 0.9999
+ 10	44.42	80.74	50.30	81.06	**11.53**	26.56–76.51	68.48–141.3	29.39–82.09	70.91–138.5	0.0007*/r = 0.48	> 0.9999
+ 5	37.18	83.34	34.50	83.43	−1.57	20.95–82.13	71.08–113.7	21.36–81.72	71.54–116.3	0.6986/r = 0.18	0.1092
−5	34.88	77.38	42.74	76.55	10.49	21.23–73.93	63.49–115.3	21.39–73.62	63.95–119.1	0.0238/r = 0.31	> 0.9999
−10	31.85	79.89	30.82	79.49	−2.42	19.94–75.51	65.07–108.4	20.60–75.52	65.27–105.3	0.3948/r = 0.15	> 0.9999
−20	31.45	69.73	56.63	70.71	11.78	18.09–76.68	56.65–94.45	18.31–92.59	57.47–88.32	> 0.9999/r = 0.14	> 0.9999
**t-stellate**											
+ 50	24.38		25.97		−1.41	19.63–28.70		20.93–29.86		0.4515/r = 0.42	
+ 20	28.11		30.13		2.21	21.78–32.18		21.98–35.04		0.0413/r = 0.58	
+ 10	26.12		25.56		−3.13	20.48–30.25		19.88–31.55		0.9999/r = 0.24	
+ 5	19.74		24.84		14.09	15.79–25.21		19.20–29.52		0.0140/r = 0.63	
−5	16.29		18.65		0.32	14.48–21.91		14.76–23.08		0.3416/r = 0.44	
−10	15.03		25.39		39.07	12.41–19.36		17.10–29.55		0.0140/r = 0.63	
−20	18.03		28.69		29.29	13.34–20.39		15.77–32.15		0.0273/r = 0.60	

**Figure 5:**
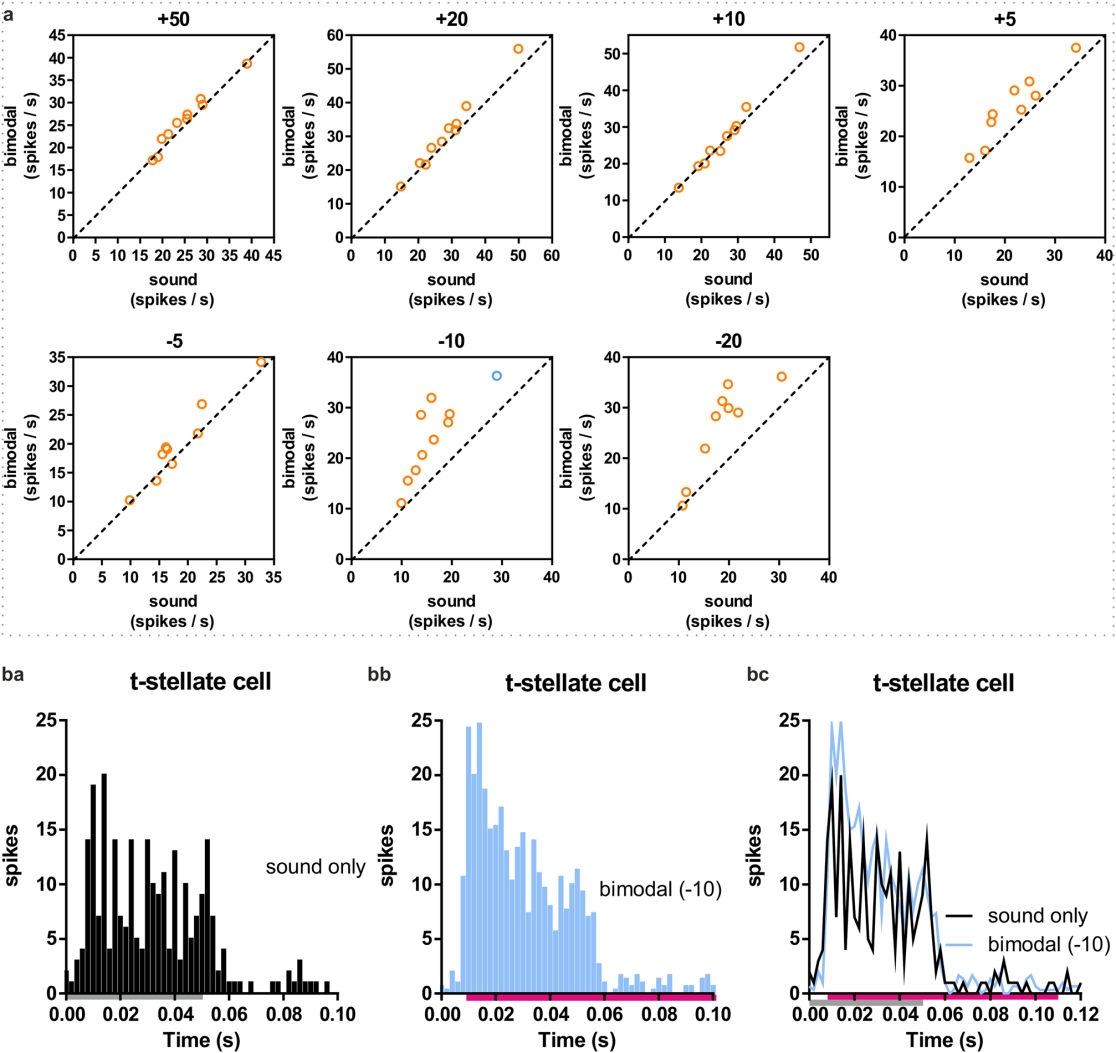
Bimodal response in t-stellate cells in VCN. (**a**) Bimodal compared to only sound stimulation. Each of the protocols (+ 50, + 20 etc.) plotted separately. Each dot is one spike-sorted unit. The dotted line represents a linear regression line with slope 1 (y = x). Blue circle is the example cells from *ba-bc*. (**ba-bc**) The PSTH of a representative cell is plotted for the − 10 protocol (blue dot in the panel “−10”). (**ba**) The PSTH for only sound. (**bb**) The PSTH for the bimodal stimulation. (**bc**) The overlay of the PSTHs from *ba* and *bb*. The grey bar shows the duration of the sound stimulation and the pink bar the duration of whisker stimulation. Sum of 500 stimulations each for sound and bimodal.

**Figure 6:**
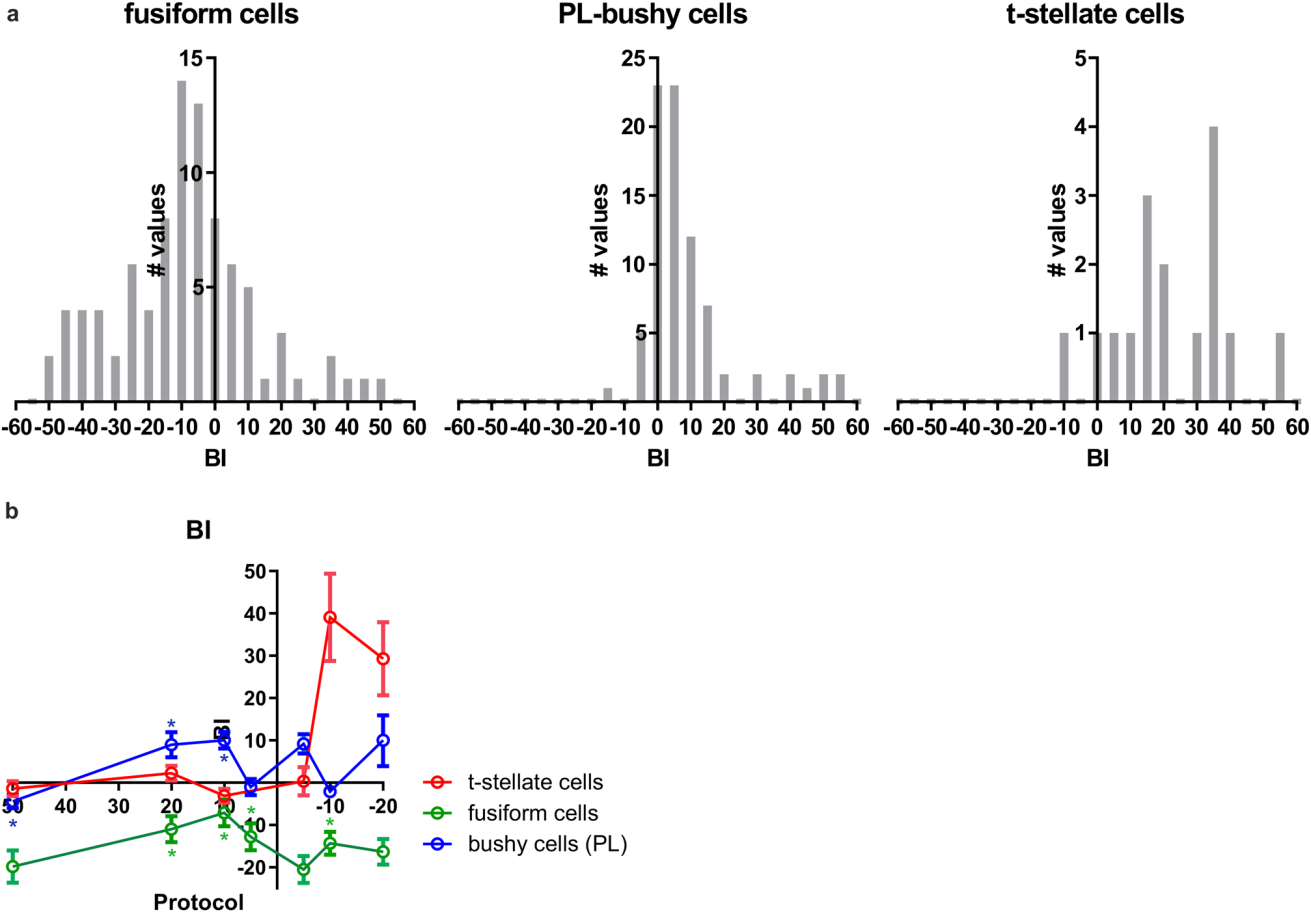
Bimodal integration index. (**a**) Histograms of the BI (bimodal integration index) for bimodal protocols where the spiking was increased compared to only sound stimulation. In fusiform cells, and primary-like bushy cells the BIs for the + 20 & + 10 protocols are plotted. For the t-stellate cells the − 20 and − 10 protocols. Two values for each cell, one for each of the two protocols. (For the t-stellate plot, four values with a BI > 60 are not included in the plot.) (**b**) The BI (mean ± SD) for all recorded protocols in fusiform cells, primary-like bushy cells and t-stellate cells. * in the corresponding colour mark the protocols where there was a significant difference in spiking activity between bimodal and sound stimulation.

In t-stellate cells (n = 10), the whisker alone stimulation induced a short-lasting (~ 20 ms) increased spiking (Fig. 2f), hence, when the whisker stimulation preceded the tone, the whisker stimulation did not influence the sound evoked response (Fig. 5, Table 2). Although the bimodal response was not significantly larger, when averaging over the sample, in some cells the brief burst of whisker-evoked activity could increase the bimodal response. This effect is corroborated by the relatively large BI (for the − 20 and − 10 protocols; Fig. 6).

## Discussion

In the dorsal and ventral cochlear nucleus, the sensory-evoked spiking activity to sound, whisker deflections and bimodal (whisker and sound) stimulation was investigated using in vivo electrophysiology in anaesthetized mice.

Whisker deflections evoked increased spiking activity above spontaneous rates in the cochlear nucleus. The effect was most prominent in DCN fusiform cells, with a smaller, transient response in VCN t-stellate cells. Fusiform cells receive multisensory information via granule cells, whereas for t-stellate cells a multisensory input pathway remains to be investigated. In VCN bushy cells, however, the sample average did not increase significantly, although in some cells (Fig. 2d) there was a clear increase in spiking with whisker deflections.

The cochlear nucleus is primarily involved in sound processing, thus the pairing of whisker stimulation with sound was investigated. Pairing sound stimulation with whisker deflections increased the sound-evoked responses in fusiform cells. The effect was dependent on the temporal relationship between the whisker defection and the sound stimulation. An optimal range was found to be when the whisker deflection began 20 ms before the sound onset. The average bimodal increase was, however, smaller than the sum of the unimodal activity (sound plus whisker) (mean BI ≈ − 9 (average from the + 20 and + 10 protocols; Fig. 6). The variability in the bimodal effect could indicate that multisensory integration is more prominent in certain areas and/or that some cells were less activated by the stimulation parameters used. In VCN, cells were classified based on the PSTH pattern as either primary-like (PL) or primary-like-with-notch bushy cells (PLN), the former thus putatively being spherical bushy cells and the latter globular bushy cells^30^. Globular bushy cells are important for encoding interaural level differences, whereas the spherical bushy cells play a role in encoding interaural time and level differences^31^. Based on a limited sample (n = 7) in contrast to PL cells, the sound evoked response of PLN cells is not modulated by whisker deflections. Whereas in PL cells there was a bimodal enhancement (average bimodal increase, that was larger than the sum of the unimodal activity; mean BI ≈ 11 (average from + 20 & + 10 protocols); Fig. 6). In the t-stellate population, it’s noteworthy that the whisker deflection alone caused a transient initial burst (Fig. 2f), and when the whisker deflection preceded the tone, the bimodal activity was not significantly increased compared to only sound. Delaying the whisker deflection relative to the tone increased bimodal activity, because the whisker evoked burst was preserved in the bimodal response (Fig. 6b).

Anatomical data show that DCN receives input from the somatosensory system via dorsal column nuclei and the spinal trigeminal nuclei that project to the granule cell domain^9,10,13,32^. The DCN thus plays a central role in early multi-sensory integration of sound and tactile stimuli. The spinal trigeminal nuclei also mediate sensory information from the whiskers^33^. In rodents audiotactile interactions in the cochlear nucleus have been less examined^34^. The DCN is involved in auditory spatial perception in the vertical plane and suppression of self-generated signals^20^. The interactions of sound with the head creates different acoustic spectra depending on the angle of the incoming sound. These difference in the acoustic spectra enable the DCN to determine the sound location with respect to the ear^35^. Knowing the position of the ears also necessities integrating head position. Head position is determined by proprioceptive inputs from the neck. Breathing and sniffing, activities that self-generate sound^21^, are both linked to whisking^36^. It is thus possible that whisker deflection per se is not the most important input to DCN, but rather the information on head position inherent in the sensory signal evoked by whisker movements. Furthermore, sound produced when the whisker touches an object is another source of information that could be used for multisensory integration. Hence, whisker input might provide information on subtracting self-generated sounds^20^. In guinea pigs, fusiform cells have been shown to integrate auditory with somatosensory inputs evoked by stimulation of face and neck muscles^3,16^. This type of somatosensory stimulation induced D-stellate cell inhibition, with the degree of inhibition increasing or decreasing depending on the temporal relationship between the somatosensory and the auditory signal. In the present experiments, when whisker activation preceded the sound stimulation, the bimodal response was larger compared to only sound stimulation. Under this condition it is therefore possible, that the degree of D-stellate inhibition to fusiform cells was very small.

Interactions between the somatosensory and auditory systems has been shown in humans^37–39^, cats^22^ and guinea pigs^15,40^. It remains to be investigated to what degree similar multisensory integration is important in mice and rats for tactile information processing based on whisker inputs. One hypothesis is that, similar to that found in humans^39^, depending on the frequency band, sound can enhance or decrease the perception of surface textures. Depending on the texture discrimination task the performance might thus be better or worse when it is accompanied with sounds of different frequencies.

## Materials and methods

The study was carried out in compliance with the ARRIVE guidelines and were approved by the Institutional Animal Care and Use Committee at the University of Michigan (IACUC; Protocol #009,202). We confirmed that all experiments were performed in accordance with IACUC guidelines and regulations.

### Surgery Procedures

In vivo electrophysiology recordings in the DCN and VCN were performed in eight male C57/BL6 mice (20–24 g) (JAX stock #000,664, Jackson Laboratories, Bar Harbor, ME). Animals were anaesthetized with an intraperitoneal injection of ketamine (97 mg/kg)/xylazine (16 mg/kg) and the body temperatures were kept constant (37 °C) using an automatically controlled heating pad. Additional anaesthetic (2–5% of the original dose) was administered approximately every hour after performing a pinch to check the paw reflex. After a local lidocaine (2% w/v) injection in the skin of the head, animals were placed in a stereotaxic frame secured with hollow ear-bars (Kopf Instruments) to deliver sound to the left ear. An incision was made in the skin, followed by a craniotomy over the left CN (anterior–posterior − 1.3 to − 1.6 mm (from lambda); lateral to midline 2.0 to 2.25 mm. Extracellular recordings were obtained with 32-channel silicon electrode arrays (A2 × 16–10 mm–50–500–177; NeuroNexus Technologies, MI, USA) in the DCN (~ − 1.3 mm anterior–posterior from lamda); ~ 2.1 mm lateral to midline; depth ~ 4750 µm (from skull)) and VCN; (~ − 1.5 mm from lamda); ~ 2.25 mm lateral to midline; depth ~ 5250 µm (from skull)) using the RZ2 multichannel acquisition system from Tucker-Davis Technologies (FL, USA). The raw signal (acquired at 25 kHz) was bandpass filtered between 300–5000 Hz and signals that exceeded the background noise with at least 4 SDs were detected as spikes. Recorded units were sorted offline into single-units with a customised, semi-automatic MATLAB algorithm via *k-*means clustering of *n* principal components (*k* and *n* were user-specified) of peak-amplitude aligned waveforms. Furthermore, spike-sorted units were analysed (Neuroexplorer, Nex Technologies, USA) with cross- and autocorrelation to ensure that they were separate units (Supplementary Fig. S2). Cross-correlation was done with spike-sorted units in adjacent channels. The refractory period in the autocorrelogram was estimated as the peak from the hazard function. The refractory period for fusiform cells in DCN was 9.8 ms ± 1.6 ms (mean ± SD; n = 45 spike sorted units). For bushy cells in VCN the refractory period was 8.4 ms ± 1.77 ms (mean ± SD; n = 49 spike sorted units; primary-like-with-notch (PLN): 8.3 ms ± 1.5 ms (7 units), primary like (PL): 8.4 ms ± 1.8 ms (42 units) and for t-stellate cells in VCN it was 9.0 ms ± 0.89 ms (mean ± SD; n = 10 spike sorted units). After the experiment mice were euthanized by i.p. injections of sodium pentobarbital (Med-Pharmex Inc., Pomona, CA, USA). The brains were removed and fixed in 4% paraformaldehyde. After dehydrating the tissue in 30% sucrose, the brains were sectioned in 30 μm slices, coverslipped and observed under epifluorescence (Leica, DMLB) to visualize the electrode tract.

### Auditory Stimulation

Experiments were performed in a double-walled sound shielded chamber and acoustic signals were generated by the RX8 DSP hardware from Tucker-Davis Technologies (TDT). Sound stimulation was 50 ms broadband noise bursts (200 Hz–20 kHz) with 2 ms rise/fall times presented unilaterally through a closed, calibrated earphone to the left ears. 500 repetitions were presented at 80 dB SPL. The response latency was calculated as the timepoint when the spike rate in a 1 ms bin after stimulus onset was higher than the mean ± 2 SD calculated from 50 ms preceding the sound stimulation.

### Somatosensory Stimulation

To stimulate the whiskers ipsilateral to the CN recording side a custom-built magnetic whisker stimulation system was used. This enabled whisker deflection in the absence of sound. The whiskers were lightly covered with magnetic paint (k03151000, Krylon) and an electromagnet coupled to an isolated electromagnetic driver was placed close to the whiskers. Signals to drive the magnet were generated using the TDT Synapse software. The whiskers were deflected (ramp and hold) 1000 times at 5 Hz with a 100 ms duration pulse. The whiskers were actively moved using the magnet, in the rostral direction (i.e. protraction). To ensure that sound-driven responses in the CN were caused by whisker movements, and not by other tactile or auditory cues, a control experiment with magnetic stimulation was performed by omitting the magnetic paint, and thus not eliciting movement of the whiskers when the magnet was activated. In this control experiment no effect of magnetic stimulation was observed on the spontaneous or sound-driven responses in fusiform cells (Supplementary Table S1). The time window used to calculate the spontaneous activity was 50 ms before the stimulus onset. The time window to calculate the whisker response was from stimulus onset to 50 ms (for the t-stellate cells it was only 25 ms; the t-stellate response was only on initial burst) after stimulus onset. The whiskers ipsilateral to the CN recording site (left side) were deflected. The response latency was calculated as the timepoint when the spike rate in a 1 ms bin after stimulus onset was higher than the mean ± 2 SD calculated from 50 ms preceding the whisker stimulation. The delay from magnet onset to whisker movement (~ 10 ms) was subtracted.

### Bimodal Stimulation

To study multisensory integration in DCN and VCN the acoustic stimulation was combined with the whisker stimulation. A bimodal stimulation protocol was used based on previous in vitro^41^and in vivo^42^ studies on spike timing dependent plasticity in DCN. Seven different protocols with varying delays between the sound and whisker stimulation were used: + 50 ms, + 20 ms, + 10 ms, + 5 ms, − 5 ms, − 10 ms and − 20 ms. The prefix “ + ” means that the whisker stimulation starts before the auditory stimulation and the “−” that the whisker stimulation starts after the auditory stimulation. The different protocols were repeated 500 times and the spiking output compared to 500-times acoustic stimulation alone. The order of the protocol was randomized between experiments (Supplementary Table S2). The time window used to calculate the bimodal response was ~ 50 ms for protocols + 50, + 20, + 10 and + 5. When the whisker stimulation started after the sound the time window was reduced. For the − 5 protocol the time window was ~ 45 ms, for the − 10 protocol the time window was ~ 40 ms and for the − 20 protocol the time window was ~ 30 ms (the exact time varied 1–2 ms depending on the stimulus artefact). To compare the bimodal response to the unimodal responses (only sound or only whisker deflections) the percentage change was calculated as a bimodal integration index $BI\equiv \left[\frac{Bi-S-W}{S+W}\right]*100,$ where *Bi* is bimodal, *S* is sound, and *W* is whisker response in spikes/s^21,43^. BI > zero, is called a bimodal enhancement, meaning that the bimodal response was larger than the sum of the unimodal response.

### Statistics

The raw data (spikes/s) did not pass normality tests (neither the D'Agostino & Pearson omnibus normality test, nor the Shapiro–Wilk normality or the Kolmogorov–Smirnov normality test); the data was right-hand skewed–relatively few data points with a high spike rate and many with low spike rate. Furthermore, the sample variances in each group were not equal. Thus, the groups were compared with individual non-parametric tests (Wilcoxon matched-pairs signed rank test, exact p-value), with p-values corrected for multiple testing using Bonferroni correction. There is one comparison per row/condition (sound vs. whisker deflection + sound); thus, the number of comparisons per family was seven, one for each protocol (+ 50, + 20 etc.). In the tables (Tables 1 and 2) the p-values from the individual Wilcoxon matched-pairs signed rank test were multiplied by seven. Unless stated otherwise the median and interquartile range (IQR) is plotted. The interquartile range is the difference between the third and first quartiles. Due to the expected variability in the response of different cells to bimodal stimulations, the experimental unit was each individual cell, rather than averaging cells from each animal. To reduce the Type 1 error rate, taking into account the large sample size when using the individual cells as the experimental units, the significance level was set at p < 0.01. The correlation coefficient *r* was calculated as a measure of the effect size: $r=\frac{z}{\sqrt{2*n}}$. Z is the z-score, n = number of single units, 2n = the total number of observations, including the cases where the difference is zero.

## Supplementary Information

Supplementary Information
