# Supplementary material for: Audiotactile interactions in the mouse cochlear nucleus
Source: Sci Rep. 2021 Mar 25;11:6887. doi: 10.1038/s41598-021-86236-9 (PMC7994829; doi:10.1038/s41598-021-86236-9)
Supplement: Supplementary file 1 — Supplementary Information [file 41598_2021_86236_MOESM1_ESM.pdf]

Supplementary Information

Audiotactile interactions in the mouse cochlear nucleus

Josephine Ansorge[1], Calvin Wu[2], Susan Shore[2,3,4], Patrik Krieger[1 *]

[1]Faculty of Medicine, Department of Systems Neuroscience, Ruhr University Bochum, Bochum, Germany

[2]Kresge Hearing Research Institute, Department of Otolaryngology, [3] Biomedical Engineering, [4]Molecular and integrative Physiology, University of Michigan, Ann Arbor, Michigan, USA

[*]Corresponding author

Patrik Krieger
Dept. Systems Neuroscience
Ruhr University Bochum
Universitätsstraße 150
D- 44780 Bochum
Germany

Supplementary Table S1

fusiform cells	Median (spikes / s)		IQR		p-value (Bonferroni corrected)
Protocol	sound	magnet + sound	sound	magnet + sound	
+50	20.60	20.53	17.60 – 45.10	17.00 – 43.40	0.9999
+20	22.47	23.75	9.00 - 33.44	8.85 – 33.03	0.9999
+10	22.75	22.89	10.71 – 36.97	11.33 – 37.17	0.7658
+5	23.03	24.38	11.75 – 40.60	10.08 – 38.93	0.9842
-5	23.00	24.61	9.18 – 41.94	9.39 – 42.29	0.9999
-10	23.50	23.33	8.96 – 39.44	7.92 – 41.25	0.9999
-20	24.47	24.93	15.00 – 28.50	13.27 – 26.83	0.8666

Negative control experiments to show that the magnet stimulation per se did not evoke a response

The magnet stimulation was used without magnet paint on the whiskers. The spiking activity was not changed when the magnet was used without causing a whisker deflection. Wilcoxon matched-pairs signed rank test, and Bonferroni correction. Data from eight fusiform cells.

Supplementary Table S2

Experiment 1	+20	+50	+5	+10	-5	-10	-20
Exp. 2	+10	+20	+5	+50	-10	-5	-20
Exp. 3	-20	+20	+10	-10	-5	+5	+50
Exp. 4	-5	+10	+20	+50	+5	-10	-20
Exp. 5	-5	-10	-20	+5	+10	+20	+50
Exp. 6	+50	+20	-10	+10	-5	+5	-20
Exp. 7	-5	-10	-20	+5	+10	+20	+50
Exp. 8	+50	+20	+10	+5	-5	-10	-20

The protocol order was changed between all eight experiments

In experiment one the bimodal stimulation was first tested with a "+20" protocol, i.e. whisker stimulation preceded the broadband noise (sound) stimulation by 20 ms. Followed by the "+50" stimulation et cetera. For each cell, each of the seven protocols was tested 500 times (i.e. 500 bimodal stimulations compared to 500 sound only stimulations).

Supplementary Figure S1

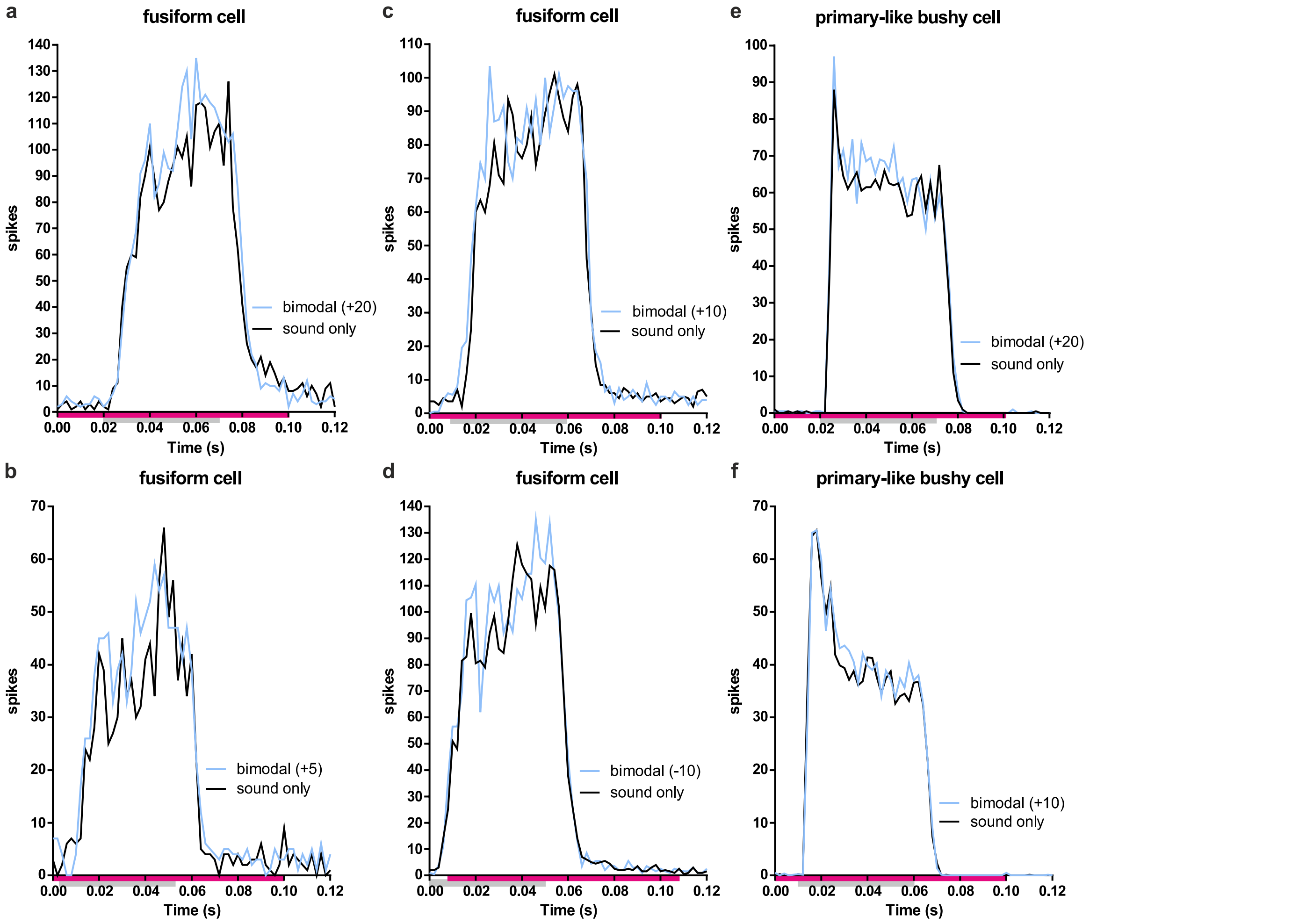

Example PSTHs from a fusiform cell and a primary-like bushy cell for all significant stimulation protocols

(**a-d**) Example PSTHs from four different fusiform cells for the +20, +10, +5 and -10 protocols, respectively. Sum of 500 stimulations each for sound and bimodal. (**e-f**) Example PSTHs from two primary-like bushy cells (PL) for the +20 and the +10 protocol, respectively. Sum of 500 stimulations each for sound and bimodal. The pink bar shows the duration of the whisker stimulation, and the grey bar the duration of sound stimulation.

Supplementary Figure S2

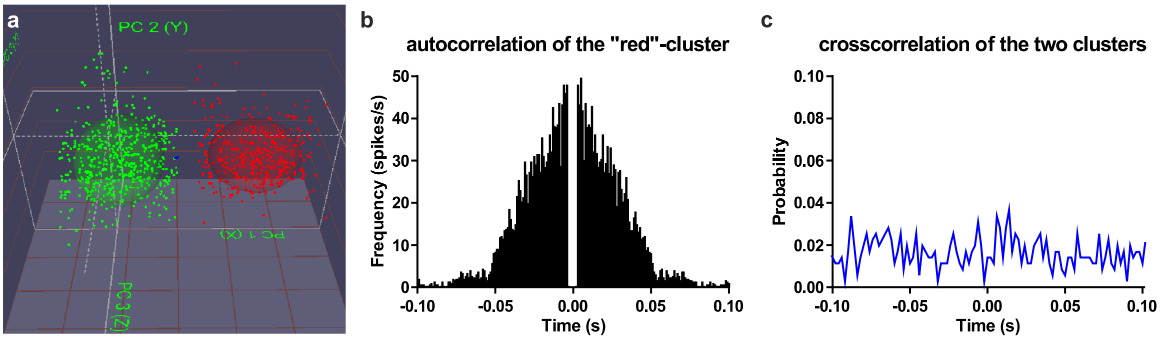

Spike sorting

(**a**) 3D principal component analysis showing two separate clusters representing two spike-sorted unit recorded from fusiform cells. (**b**) Spike-sorted units were analysed with autocorrelation to verify the spike sorting. An example of autocorrelation for the "red" cluster. The "gap" around zero shows that no false positives were included. (**c**) The flat cross-correlogram shows that the spike-sorted units were correctly classified as two different units.
